# On the Combination of Tin and Gold as a Filling Material for the Teeth

**Published:** 1888-01

**Authors:** W. D. Miller, St. George Elliott


ARTICLE V.

### ON THE COMBINATION OF TIN AND GOLD AS A FILLING MATERIAL FOR THE TEETH.

BY W. D. MILLER, M. D., PH. D., &C.

[Being a series of Lectures delivered in the Institute and translated from the German by Miss St. George Elliott.] *

*Gentlemen*:—You have all, no doubt, time after time, felt that among the many materials which nowadays are used for stopping teeth not one possesses the qualities of an ideal filling substance. You have, therefore, tried to overcome the difficulty as far as possible by choosing for each separate case that material which has the greatest number of the wished-for qualties, or, to speak more correctly, that material which has the fewest objectionable qualities.

You will also readily acknowledge that in many instances the question "With what shall I fill this cavity in order to obtain the best result?" is exceedingly difficult to answer and is imperfectly solved by the choice of that material which, in the opinion of the operator, *comes nearest* to what is desired.

Under these circumstances, it is evident that, through the introduction of a new and good filling material, the practical value of dentistry is greatly enhanced.

The material to which I wish to call your attention to-day, a combination of tin and gold, seems to me in many cases to very materially diminish the difficulties of the operation for both patient and operator, and to render possible the introduction of a more permanent fillling than could be obtained by other means.

It is now about 25 years since the late Dr. Abbot, of

* At the request of the author, a number of changes made in the text, also the number of illustrations reduced to fifteen.

Berlin, first made use of the combination of tin and gold foil as a filling material; but although the Abbot method occasionally found an enthusiastic follower—I mention here only the names of my colleagues Drs. Jenkins, of Dresden, and Sachs, of Breslau,—it has only been in the past few years that a general interest in this important topic has been awakened, and this as a result of the many discussions in the different dental societies, and also the communications in the professional journals. This may be accounted for partly by the fact that the superiority of this substance was not well enough known, and partly by the circumstance that many, for fear that there would be an injurious electrical effect, did not dare to insert two different metals in the same cavity. There are still, in fact, at the present day practitioners who have this groundless fear of electrical disturbance. I have even met with cases in which dental practitioners have insisted upon removing such mixed fillings, under the supposition that they would necessarily injure the teeth, notwithstanding the fact that these teeth had, by the use of tin and gold, been kept for years in excellent conditton.

For this reason it is thought to be in place, in connection with the tin gold filling, to point out the electrical processes which may take place in filled teeth in the human mouth. As is well known, Bridgeman, in a prize essay, has endeavored to trace back to electrical action the origin of dental caries. Accorcing to his view, there are electrical currents between the different parts of the teeth, for instance, between enamel and dentine, enamel and cement, dentine and cement, &c., by means of which the mass of the tooth is destroyed in pretty much the same manner, through local currents, as is a piece of impure zinc in dilute sulphuric acic. A theory of dental caries, so poorly supported by facts of experiment and experience, produced no particular impression. It was different, however, with the theories of Chase and others. They succeeded by means of experiments, which to my knowledge have never been published, in constructing a series in which the difference of potential between the different stopping materials and dentine is shown. This series is as follows:—

| | |
|---|---|
| Electro-negative | Gold |
| Amalgam | Tin |
| Gutta percha | Dentine |
| Oxychloride of zinc | Electro positive. |

Each being E. N. to all that follow, and E. P. to all that precedes.

The result is that the greatest E. M. F. is generated by a combination of gold and dentine; the combination of amalgam and dentine furnishes a smaller E. M. F.; a still smaller that of gutta-percha and dentine. Based upon these and other experiments, Chase concluded that a tooth filled with gold would soon show a reappearance of caries upon the edge of the cavity, as a result of the strong current existing in all places where acid fluids in the mouth constantly wash the filling and the neighbouring tooth-substance. A tooth stopped with amalgam is less affected by this electro-chemical process, still less is one filled with tin—the proportion being 100, 67 and 50—while the tooth which is stopped with oxychloride of zinc, becoming electro-negative by contact with this material, remained secure against the action of acids, which are likewise electro-negative.

Chase also succeeded in proving (?) the truth of this reasoning by means of experiments. He procured several pieces of ivory of equal size and the same form, and bored a hole in each piece, filling these cavities with various filling materials. After these had been exposed to the action of an acid for a week, he found they had diminished in weight in the following proportion:—

| | | |
|---|---|---|
| The piece filled with | Gold - - | 0.06 |
| " " | Amalgam - - | 0.04 |
| " " | Tin - - - | 0.03 |
| " " | Gutta-percha - | 0.01 |
| " " | Wax - - | 0.01 |
| " " | Oxychloride of Zinc | 0.00 |

Reasoning from these results, Chase came to the conclusion that among all the filling materials in use gold is the worst, and that every tooth filled with metal is a galvanic battery, which becomes active as soon as the surrounding fluids have an acid reaction. Practitioners of good standing, determined by the experiments of Chase, entirely gave up the use of gold as a filling material. Under such circumstances, it is easy to understand why it was thought unwise to place a mixture of two metals in the same cavity. It was believed, in spite of the non-existence of any satisfactory proof, that, if one metal had such a disastrous effect upon a tooth, a combination of two metals would increase the danger of injuring certainly two-fold, if not in greater ratio. Practical experience teaches us the fallacy of this conclusion, and indeed Chase's experiments and results were of a so surprising a nature that other dentists were immediately incited to repeat the same experiments, and, so erroneous, that the errors were at once made apparent. I, myself, five years ago, began two series of experiments, with the object of finding out:—First, whether an electrical current can exist between dentine and metal; second, whether the rapidity with which dentine is affected when laid in diluted acid is dependent in any wise upon contact with a filling material.

These experiments were made in the laboratory of Du Bois-Reymond, and the results were published partly in the *Deutsche Medizinische Wochenschrift*, and partly in the *Dental Cosmos*, from which I make the following extract:—

"What electrical actions take place when we bring a living tooth into contact with metal? It has been proclaimed that every tooth filled with metal is a galvanic battery, which becomes active as soon as the surrounding fluids have an acid reaction (Chase). This statement does not, however, rest upon any experimental facts. So far is this from being the case that its advocates openly admit that nobody has yet been able to detect this galvanic action, still less to measure the same.

"Regardless of these views, I maintain that there certainly are electrical currents to be found in the mouth when it contains teeth filled with metal. These currents do not, however, exist between the filling and tooth substance in such a manner that the latter can be compared to one of the plates of a galvanic element; but the electrical processes which really take place in the mouth owe their presence wholly to the heterogeneity of the metallic fillings.

"On the surface of every filling, even of one of pure gold (when, as is always the case in practice, the filling is not throughout of a uniform density), electrical currents will be generated which flow between the denser and less dense points. But since all of these currents do not tend towards the margin, no injury to the tooth may be feared."

"When two fillings composed of different materials come in contact in the same tooth, or in any neighbouring teeth, there follows a current which is directed through the mouth and fluids of the teeth from the more oxydizable (electro-positive) towards the less oxydizable (electro-negative) metal, and this, working electrolitically upon the fluids of the mouth, may possibly produce deleterious results. Through this, current acids are produced upon the surface of the electro positive metal which may attack the tooth around the edge of the filling. This action seems, however, almost entirely to cease as soon as the surface of the positive pole becomes oxydized; in short, practice has not yet proved this process in any wise injurious to the teeth.

"Electrical currents also appear in the mouth when metallic clasps surround teeth in which are metallic fillings, or whenever clasps of baser metal are placed on a gold plate. These currents are naturally weak; nevertheless, through them, free acids may be developed on the clasp, and these in course of time may obviously greatly damage the teeth with which they come in contact.

"Electrical currents are therefore found in teeth:

"*a.*—When a metal plug is not of the same density throughout.

"*b.*—When two metallic fillings of different materials, or a metallic filling and a metallic clasp, come in contact.

"*c.*—When a plate is composed of different alloys.

"Another question in this connection concerns the electric conductivity of tooth substance.

"Now it is true that the ingredients of which dentine is composed are already non-conductors, but, nevertheless, I tested the conductivity (or non conductivity) of dead dentine by the following experiments. A section of dentine cutting the tubules at right angles, 3 100 millim. in thickness, was enclosed in a circuit consisting of three Siemen's cells, and the coils of a mirror galvanometer of 16,000 turns and a resistance of 5,000 Siemen's units. When the circuit was closed the mirror did not show the slightest deflection. The piece of dentine was in this experiment inclosed between the ends of two wires 1.9 millim. in diameter under a pressure of 3 gr. per square millimeter.

"This experiment was then varied by placing three similar pieces in the circuit, in such a manner that the surface of contact between the wires and the tooth-substance would be enlarged three-fold. The deviation still remained nil, showing the resistance to be infinitely great.

"When one pole of an electrical battery of four Siemen's elements is placed on a tooth filled with metal and the other pole on a second filling or on the gum, it is distinctly felt that the circuit is thereby closed. This fact has given rise to the belief that dentine is a conductor; now this, as has been shown, is not true, the apparent conductivity being due to the fluids which are contained in the dentinal fibrils, and the pulp canal. In this manner the porous cylinder of a galvanic element, naturally a non-conductor, is transformed into an excellent conductor, as soon as it becomes saturated with the battery solution; also a silk thread becomes a conductor as soon as it is moistened with a conducting liquid; so, too, a glass tooth whose canaliculi, pulp chamber, &c., are filled with a salt solution would readily transmit an electric current. We cannot on this account, however, consider these substances as conductors, they being notorious non-conductors, nor could they under any circumstances be used as one of the generating plates in an electric cell. Through this infinite resistance of the tooth-bone every possibility of an electric current between tooth-bone and filling is excluded, just as it would be if the basis substance of the tooth were composed of any other non-conductor; for example, glass."

I also repeated Chase's experiments, using pieces of ivory as well as dentine from human teeth and from fish teeth, and filling or bringing these into contact with various stopping materials, finally allowing them to remain for a certain time in a solution of acid. I was convinced, by a series of experiments (*Dental Cosmos*, 1881, page 91), that it makes not the slightest difference in regard to the rapidity of the decrease in weight with what the pieces are filled or whether they are, in fact, filled or not.

It is simply inexplicable how Chase attained his results, particularly how he discovered that a piece of dentine stopped with oxychloride of zinc becomes negative and consequently resists the action of the acid—in other words, is not affected at all by it.

Dry tooth-bone is, like all other dry organic substances, a non-conductor, and opposes as such as infinitely great resistance to the passage of galvanic currents. Living tooth-bone is, it is true, a conductor, but only in so far in that it is saturated with liquids. Tooth-bone cannot be used as one of the plates (poles) in a galvanic element any more than can a piece of clay; no current can therefore exist between tooth-bone and filling, nor can the affinity of tooth-mass for acids be either heightened or reduced by any filling.

The above applies equally well to a tin-gold plug as to that of any other metal. In the tin and gold filling the two metals are about equally distributed; this state produces on the surface of such a stopping a large number of indefinitely small electrical currents, which bring about a very intimate, and as yet not quite clearly defined, connection between the two metals, but which have no effect (theoretically as well as practically) upon the teeth.

After these introductory remarks, which I have deemed necessary to a right understanding of my discourse, I take pleasure in giving you some directions regarding the preparations of the material and of the cavities in which it is to be inserted.

THE PREPARATION OF MATERIAL AND OF CAVITIES.

When I wish to make a filling of tin and gold, I lay a leaf of No. 4 non-cohesive gold-foil (for this purpose I always use Abbey's) upon a leaf of No. 4 "extra tough" tin foil. These leaves I cut now into from two to four strips, according to the size of the cavity I wish to fill. For extremely large stoppings I sometimes use an entire leaf. I then twist these strips with the fingers in a loose roll, which I occasionally use whole, but generally divide into pieces of from 4 to 12 mill. in length, as the depths of the cavity requires. It is a mere matter of taste whether the tin or gold is placed on the exterior of the roll; it is not a matter of equal indifference, however, whether the tin or gold rolls are in the form of a solid rope or only a loose roll. The more compact it is, the firmer are the cut pieces and the greater the difficulty in inserting the separate bits. The tin and gold is rolled at first towards the right, until it becomes a moderately firm rope which is then unrolled towards the left, and becomes a crinkled twist. Personally, I prefer to have the tin on the outside, because it does not tear as easily as the gold foil. Dr. Jenkins, of Dresden, who has used the material for upwards of twenty years, chooses rather to have the gold on the outside, because, in his opinion, the colour of the finished stopping is, and remains, better, and moreover the cavity is better lighted during the operation by a gold than a tin surface; finally, because gold is less cohesive than tin. He prepares the material by placing an equally heavy piece of tin foil upon a leaf of non-cohesive gold foil, and pressing both leaves with the "foil crimpers" in such a manner that the gold is on the exterior. I shall not recommend any particular plan in regard to this, as it is quite immaterial whether tin or gold forms the outside of the roll. Instead of rolling the strips together, you may fold them; but in my opinion, the rolls are softer, more flexible, and easier to work. My father-in law, the late Dr. Abbot, who introduced this method of filling, always had the material prepared in rolls, and with the tin towards the surface—a practice which I follow and here recommend.

The cavity is prepared in the same way it would be were non-cohesive gold the filling material, but one can secure the retention of tin and gold in cavities where non-cohesive gold, on account of the flatness or unfavorable shape of the cavity, could not be used, much less cohesive gold. Tin-gold is also more easily contoured than non-cohesive gold.

Special retaining points are never required, and, in fact, would be quite useless.—*London Dental Record.*

